# A Scoping Review of Programs of Active Arts Engagement in International Medical Curricula

**DOI:** 10.5334/pme.1506

**Published:** 2025-05-19

**Authors:** Zoe Moula, Stephanie Bull, Naa Okantey, Megan Brown, Victoria Edleston, Maisie Crawford, Sandra Sawchuk, Tracy Moniz

**Affiliations:** 1 Department of Care in Long Term Conditions, King’s College London, UK; 2 Medical Education Innovation & Research Centre (MEdIC), Department of Primary Care and Public Health, School of Public Health, Imperial College London, UK; 3 Department of Primary Care and Public Health, School of Public Health, Imperial College London, UK; 4 School of Medicine, Newcastle University, UK; 5 King’s College London, UK; 6 Liaison Librarian to the Sciences, Mount Saint Vincent University, Canada; 7 Department of Communication Studies, Mount Saint Vincent University, Canada

## Abstract

**Introduction:**

Arts and humanities are often positioned as ‘additive’ to medical education, rather than ‘intrinsic’. They are also used to teach skills and perspective-taking more than utilising their transformative potential to propel personal insight and social advocacy. There is, therefore, a need for more meaningful and strategic integration of the arts in medical curricula. Existing reviews combine *active* and *receptive arts* engagement, although these methods represent different magnitudes of engagement.

**Methods:**

This review aimed to synthesise the use of *active* arts engagement in undergraduate medical curricula internationally. We searched seven databases for articles published between 1991–2024.

**Results:**

We reviewed 134 studies conducted in 27 countries (total n = 10,700). Most programs were medium-intensity (e.g., standalone modules), used visual and performing arts, and aimed to foster skills mastery, perspective-taking, and personal insight. Studies on artmaking for social advocacy were lacking, as was data about program evaluation and learner assessment. Almost all survey instruments used were unvalidated.

**Discussion:**

Studies of active arts engagement are disproportionately low compared to receptive engagement, signaling missed opportunities to leverage the benefits of the arts. Most studies were conducted in high-income countries, illuminating that lower-income countries do not have a strong voice in the knowledge exchange. To avoid devaluing the arts in medical curricula, we suggest that medical educators: a) direct attention to creative opportunities to engage students with social advocacy; b) collaborate with arts/humanities professionals and international medical educators; c) consider more meaningful and strategic integrations of active arts engagement into medical curricula, approaching them with the same rigor as other medical education programs to maximise their pedagogical potential.

## Introduction

In 2021, the largest scoping review of the arts and humanities in medical education (769 articles) showed that arts and humanities have vast potential to contribute towards skills mastery, perspective taking, personal insight, and social advocacy [1]. The review analyzed various art forms and humanities subjects, including literary arts, creative writing, film, theatre, music, and dance, specifically in the United States and Canada. The authors found that arts and humanities were often positioned as “additive” to medicine, rather than “intrinsic”, and used to teach skills and perspective taking more so than to tap into their transformative potential to develop personal insight and propel social advocacy [2]. The authors, therefore, called for critical reflection on and more meaningful and strategic integration of arts and humanities in medical education [1,2].

Following the insights and gaps identified in this review, our scoping review narrows the focus to the role of *active arts engagement* in the education of students at undergraduate medical schools—exploring this from an international perspective, beyond the United States and Canada. We used the arts and health glossary [3] to distinguish between definitions of *active* and *receptive* arts engagement. Active engagement includes overtly/directly making, performing, or creating art such as painting a picture, writing a poem, making a movie, or acting in a play. Receptive engagement includes experiencing, attending, listening, or viewing art, such as viewing a painting in a gallery, reading a poem, watching a movie, or attending a performance [3] (Table 1).

**Table 1:** Examples of active versus receptive arts engagement (adapted from Davies & Clift [3]).


**EXAMPLES OF ACTIVE ENGAGEMENT**

Visual arts: drawing, painting, sculpting, photography, mural making, pottery, knitting Literature: creative writing, such as producing comics/graphic novels, storytelling, poetry, journaling Performing arts: singing, playing musical instruments, dancing, acting, script writing, composing, jamming, busking, producing film or podcasts Digital arts: producing digital photography, filmmaking, animation

**EXAMPLES OF RECEPTIVE ENGAGEMENT**

Visual arts: visiting a gallery or museum, attending an exhibition or a fashion show Literature: reading novels, attending a book launch or a talk, joining a book club Performing arts: attending a concert, theatre, dance, or performance, watching a play, listening to music or podcasts Digital arts: watching a film, performance, or animation, viewing an e-concert or e-gallery


To our best knowledge, there is no other review on engaging in arts creation within undergraduate medical education. Existing reviews [1] combine *active* and *receptive arts* engagement, although these methods represent different magnitudes of engagement. Active arts activities offer higher levels of engagement than receptive activities [4] and tend to result “in a higher ‘arts dose’ compared to receptive methods” [5]. For instance, active participation in artmaking may require students to expose themselves, navigate and express thoughts and feelings that are difficult to articulate, and reflect on their professional identity and role as a future doctor [6].

Despite limited synthesis of evidence for active arts engagement, engagement with the arts has the potential to foster self-awareness, which in turn may help combat potential burnout among medical learners [7]. Such outcomes are especially important for medicine, given that medical students have poorer mental health compared to other students [8]. This is a result of (a) higher academic stress, expectations and competitiveness [9–10]; (b) exposure to end-of-life experiences, health risks, moral and ethical conflicts [11]; and (c) reluctancy to disclose health challenges due to stigma or concerns around career progression [12,13]. Active arts engagement has the potential to enable medical students to realize their interpersonal, perceptual, and expressive capacities, thus developing attention to nonverbal cues, connecting better with patients [14], reflecting on their professional identity formation [6,15], and supporting students to flourish [16,17].

While existing literature describes various uses of arts as pedagogical tools, less is understood about the ‘where’, ‘why’ and ‘how. Through this review, we seek to understand where, geographically, active arts engagement is discussed in relation to undergraduate medical education. Our research responds to the gap—and need—in the field to consider articles emerging from within and outside the global north, thus adding to the rising wider discourse in taking a decolonial approach where research has historically privileged the western voice. We therefore could not assume that outcomes from one geographical region (e.g., high-income countries) would be relevant across locations and cultures and sought to interrogate this in our study [18]. We also explore the learning objectives and outcomes of active arts engagement, and the methods used to evaluate programming and assess student learning. A deeper understanding of the role of active arts engagement may support more strategic and meaningful integration of arts-based curricula in medicine and advance a shared agenda for arts in medical education [1].

## Methodology

This research involved a scoping review to address the overarching question: Where, why and how is active arts engagement being used in undergraduate medical education?

To explore this question, we further asked:

- In which countries is active arts engagement being used in undergraduate medical education?
- What purpose(s) (i.e., learning objectives) is active arts engagement serving within undergraduate medical education? Are the learning objectives achieved?
- How are programs of active arts engagement being evaluated?
- How is active arts engagement integrated into curricula?
- How are learners being assessed?

We followed the methodology set out by Arksey and O’Malley [19], except for the optional consultation phase. We included articles related to *active* arts engagements in undergraduate medical education globally. We defined ‘arts’ in terms of five main forms: performing arts; visual arts, design and craft; community and cultural festivals; literature; and online, digital and electronic arts [3]. The eligibility criteria are presented in Table 2. The search strategy was based upon the exact PubMed search strategy published in the Moniz et al. [1] scoping review’s Supplemental Digital Appendix 1, with equivalencies for subject headings across databases. We searched seven databases (PubMed, ERIC, CINAHL, Cochrane Library, Web of Science, PsycINFO, EMBASE) for articles published between January 1, 1991, and December 10, 2024.

**Table 2:** Eligibility criteria.


INCLUSION	EXCLUSION

Any geographical location	No geographical exclusion criteria

Undergraduate medical education (i.e. students at undergraduate medical schools)	Postgraduate medical education (i.e. qualified medics) Combination of under- and post-graduate medical students Combinations of undergraduate medical students and students from other healthcare or health allied disciplines. However, instances where students from arts and humanities disciplines facilitated the programmes to medical students were included.

Active arts engagement, defined as overtly or directly making, performing, or creating any of the following art forms: Performing arts* Visual arts, design, and craft Literature Online, digital, and electronic arts Community and cultural festivals Examples presented in Table 1. * Performing arts includes role play and simulation. We considered role play as active arts engagement when students improvised their role in a way that is rooted in theatre. However, we did not consider simulation (i.e., working with a simulated patient or actor) as active arts engagement. Combination of active and receptive arts engagement was included because receptive engagement is often used to stimulate active engagement.	Receptive or passive arts engagement, defined as experiencing, attending, listening, or viewing any of the following art forms: Performing arts* Visual arts, design, and craft Literature** Online, digital, and electronic arts Community and cultural festivals Examples presented in Table 1. * In performing arts, simulation was considered receptive arts engagement and was excluded. ** In literature, we excluded creative writing for the sole purpose of academic publication as well as reflective writing.

Literature indexed within these databases: PubMed, ERIC, CINAHL, Cochrane Library, Web of Science, PsycInfo, EMBASE. Artciles published from 1991–2022. Articles published in English language.	Literature reviews (excluded but used to inform our scoping review). Reports and conference proceedings as they were not searched for within the original scoping review, on which this paper builds. Articles not published in English language.


**Figure 1:**
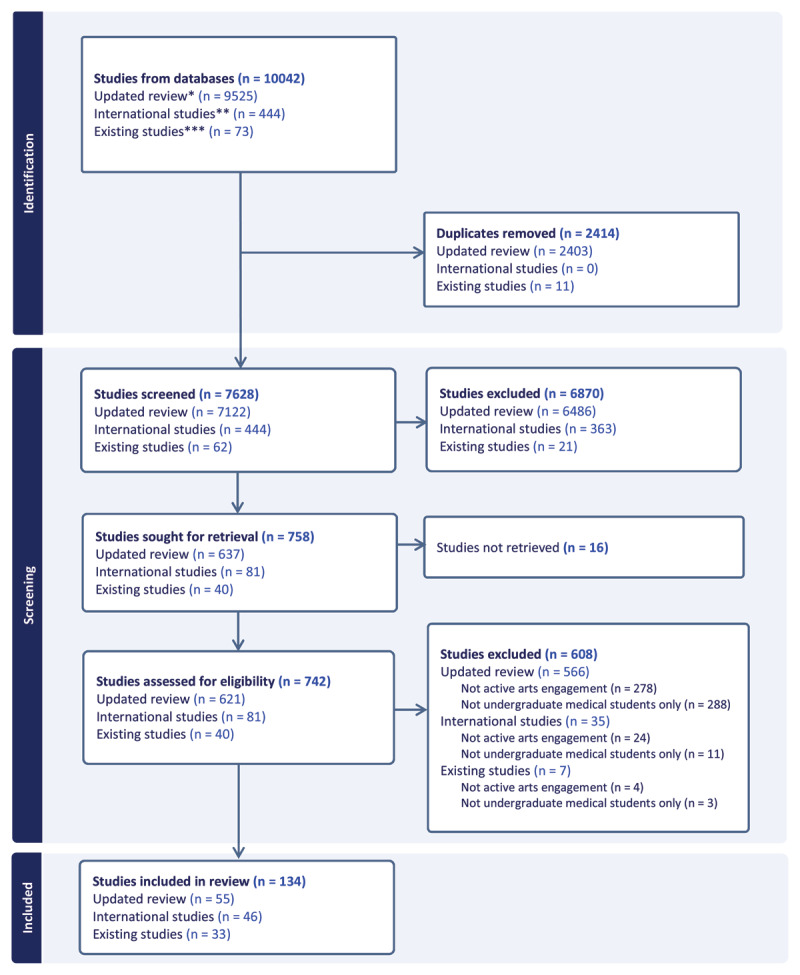
Arts Scoping Review – PRISMA Diagram. * **Updated review** includes articles retrieved from updated search from 2019-2022. Searches on CINAHL, Cochrane, Embase, ERIC, PsycInfo, PubMed and Web of Science were concluded on December 10, 2024. ** **International studies** includes articles originally excluded from previous review (Moniz, et al. [1]) due to geography. *** **Existing studies** includes articles from the original review that were identified as involving the creation of arts (Moniz et al. [1]).

We identified and screened articles in three stages. For each stage, two trained reviewers screened each record at title-and-abstract and full-text levels. Uncertainties were resolved through team discussion. First, we began with the studies identified by Moniz et al. [1] involving creating arts and rescreened them to identify those meeting our specific inclusion criteria, such as involving students studying at undergraduate medical schools and not involving mixed populations of health professions students (n = 33). Second, we screened the international studies originally excluded by Moniz et al. [1] and identified those meeting our eligibility criteria (n = 46). Third, we updated the literature by re-running the search across the seven databases to capture eligible records published from May/June 2019 (the end dates for the Moniz et al. search [1]) to December 10, 2024 (n = 55). Overall, we screened the title-and-abstract of 7,628 articles and full-text of 742 articles and included 134 studies (Figure 1).

Six authors developed, piloted, and updated the data charting form to determine if the approach was consistent with the research question and review purpose. We charted descriptive details (e.g., country, art form, research design); curricular details (e.g., program length/intensity; core/optional; instructor profile); learning objectives, using the Prism Model of four functions of arts/humanities teaching in medicine (mastering skills, perspective taking, personal insight, and social advocacy) developed by Moniz and colleagues [2]; program evaluation, using Kirkpatrick’s four levels (reaction, learning, behaviour, and results) [20]; and learner assessment.

## Results

We first describe demographic and related characteristics of the studies, and then outline the key patterns in the content.

### Demographic findings

**Countries:** The studies were conducted in 27 countries (number of articles from each country in parentheses): USA (63), UK (25), India (6), Hong Kong (4), Nepal (3), Brazil (3), Canada (3), Australia (4), Netherlands (2), France (2), Germany (2), China (2), and Greece (2). We also found one study from each of these countries: Republic of Ireland, Japan, Taiwan, Malaysia, South Africa, New Zealand, Thailand, Hungary, Spain, Turkey, Iran, Switzerland, and Finland. In these lesser represented countries, performing arts were most common (8/14 articles). Authorship affiliation was available for 130/134 articles; 114 articles involved authors from the same country and 16 involved international collaboration.

**Figure 2:**
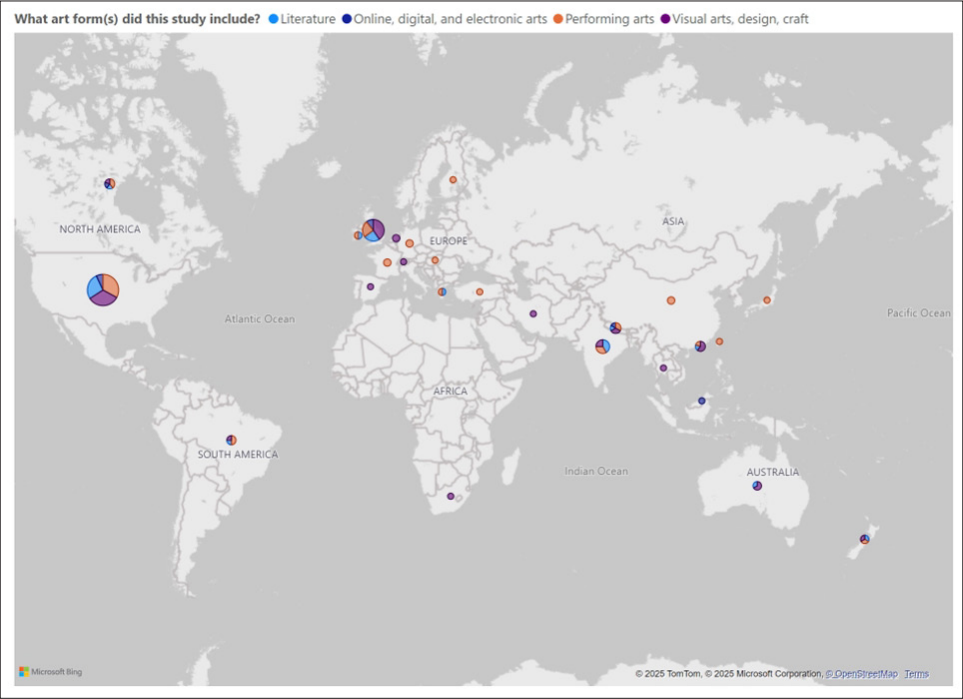
Country of study and art form.

**Types of arts:** Most studies implemented visual arts (60), performing arts (58), and literature (41). Of the literature studies, 36 involved students in poetry and creative writing and five in graphic medicine. Combining art forms was also common (30). Digital, online, and electronic arts (13) were less frequent, and design and craft and community and cultural festivals did not feature in the studies identified. In 94/134 studies, active arts engagement was the only/primary focus of the intervention (e.g., as opposed to being one among other pedagogical activities). Figure 2 illustrates the proportion of type of arts per country.

*Visual arts* included activities such as drawing, painting, photography, sculpting, art psychotherapy [21] and a global creative competition [6], as well as more specific techniques such as Kintsugi [22], mandalas [23], observational anatomy drawing and life drawing [24], mask-making [25], and medical clothing design to celebrate women in medicine [26].

*Literature* included comic-making [27], poetry [28], and writing ‘love and break-up letters’ [29] to explore medical issues and develop empathy.

*Performing arts* included common activities such as improv theater, role-playing, filmmaking, songwriting, or puppetry. More innovative practices included forum theatre (theatre that encourages audience interaction to explore social concerns) on topics such as disabilities [30] and oppression [31,32], Harry Potter-themed events [33], choreography [34], music therapy [35], and combination of performing arts [32,36]. Kuchipudi and Bharatanatyam forms of Indian classical dance were also used to interrogate ethnic and racial biases [37]. Performing arts were used to reflect on issues such as climate change [38], medical ethics [39], emotional honesty [40] or more specific topics such as dramatized headache scenarios [41], changing attitudes towards dementia [42], or breaking bad news [43].

*Digital arts* included activities such as graphic medicine [22], digital storytelling [44], and digital drawing [45].

**Study design:** Most studies were empirical (95), using mixed-methods (41), followed by qualitative (31) and quantitative (23) study designs. Of the remaining articles, many were descriptive (31), conceptual (6), and reflective or opinion-based pieces (2).

**Sample size:** The overall known sample was 10,700 medical students across 97 studies; 37 studies did not specify their sample, or this was not relevant (i.e., conceptual papers). Some studies included students from multiple cohorts, whereas others focused on single cohorts. The largest study [22] (n = 727 students) included data collected over nine years, and the smallest study involved three participants. Dropout rates and control groups were either missing or rarely reported.

**Core versus optional:** Most programming was optional or elective (85) and few were core or required components of students’ medical degree (31). One study included components of both, and 17 studies did not specify.

**Intensity:** Most studies described medium-intensity interventions (58), such as standalone programs (e.g., multiple sessions over multiple days/weeks, but not over a semester/year), followed by low-intensity interventions (40) (e.g., single event/activity), and high-intensity interventions (28) (e.g. continuous, longitudinal interventions running over a semester or integrated throughout the year). Eight studies did not specify the intensity.

### Patterns in article content

**Figure 3:**
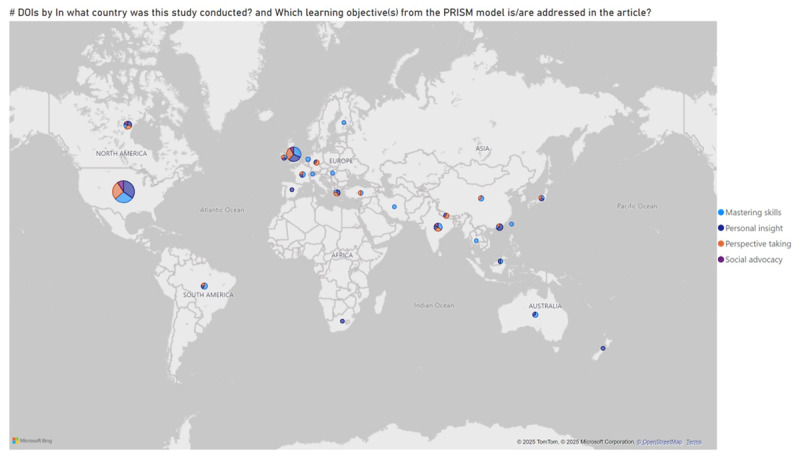
Country of study and learning objectives.

**Learning Objectives:** Table 3 presents an overview of the learning objectives of the studies, using the Prism Model framework [2]. These are also presented visually in Figure 3.

**Table 3:** PRISM learning objectives.


LEARNING OBJECTIVE	PRIMARY FOCUS	SECONDARY FOCUS

Mastering skills	68	11

Perspective-taking	50	16

Personal insight	57	23

Social advocacy	10	8


**Learning Outcomes:** Most studies (n = 117) reported evidence of improvement with respect to the learning objectives. None showed worsening outcomes. However, we relied on authors’ interpretations regarding improvements. For instance, when quantitative measures were used (n = 60), the majority (n = 55) were unvalidated with little/no evidence of psychometric characteristics. Pre-intervention data was often missing (n = 26/55), and only four studies included follow-up.

**Facilitators:** Table 4 presents information regarding program facilitators.

**Table 4:** Facilitators.


FACILITATORS

**67 studies/arts programs facilitated by medical/health faculty members** 9 members with arts training/background 6 members without arts training/backgrounds 52 articles with insufficient information regarding arts training/background

**54 studies/arts programs facilitated by medical/health faculty members alongside other instructors** 10 studies facilitated by arts/humanities faculty 21 studies facilitated by artists 3 studies facilitated by arts/humanities students 3 studies facilitated by patients 24 studies facilitated by ‘other’/unspecified instructors 15 studies facilitated by multiple instructors

**13 studies facilitated by ‘other’ instructors**


Programs facilitated by medical faculty alongside other instructors had a wider range of learning objectives, with 23% more studies with multiple objectives. Nevertheless, the proportion of studies showing improvements was only 4% higher for programs facilitated by medical faculty alongside others, compared to medical faculty alone, despite the wider range of objectives.

**Program Evaluation:** Based on Kirkpatrick’s model [20], 75 studies aligned with Level 1 (*Reaction*: evaluating program’s usefulness/relevance, learners’ engagement) and 32 studies with Level 2 (*Learning*: evaluating learners’ acquired knowledge/skills). One study aligned with Level 3 (*Behavior*: evaluating behavioral changes), and no study aligned with Level 4 (*Results*: evaluating whether outcomes were attributable to the program). Five studies aligned with multiple levels and 21 provided insufficient information.

**Learner Assessment:** Eighty studies did not use any methods to assess learners, and 39 studies did not specify. Of the 15 studies that used assessment methods, nine used summative and three used formative assessments (Table 5). More frequently, learners were assessed on the delivery of an artwork and reflection on the learning that occurred, which was often accompanied by presentations and/or exhibitions.

**Table 5:** Assessment methods.


LEAD AUTHOR (DATE)	LEVEL OF ASSESSMENT	MODE OF ASSESSMENT

Bell (2010)	Summative	Reflective portfolio (40%); Critical appraisal of research evidence (30%); Role play (40%)

Bell (2014)	Summative	Oral presentation of learning from the SSC criteria: evidence base for presentation, understanding of topic (delivery, slides, organisation), engagement with the SSC. Pass/Fail/Distinction.

Bradley (2021)	Summative	Structured literature review about graphic medicine as a genre and creating a comic. No details about assessment criteria, or outcome weighting.

Chen (2010)	Summative	Summative piece of artwork. No details on assessment criteria.

Gonzalez (2020)	Summative	Assessment based on attendance, participation, and completion of three assignments (an Implicit Associations Test and two reflections) with pass/fail grading. Formative assessment of performance in role play.

Huang (2021)	Summative	Students who role played patients in clinical skills training sessions and students who had not, undertook regular mini-CEX assessment evaluating medical interviewing, physical examination, counselling, clinical judgement, professionalism, organization and efficiency, and overall competence.

Langley (2015)	Summative	Presentation of two reflective pieces of writing, and a creative piece with written explanation.

Potash (2014)	Summative	Written extended case commentary presenting a clinical case that students had observed and discussion on how the case demonstrated the principles of patient care.

Terregino (2010)	Summative	Delivery of a creative performance to the group with pass/fail grading.

Bilella (2022)	Formative	Group presentations on clinical anatomy and pathological conditions, assessed by teacher and peer students, accounting for 30% of students’ final grade.

Card (2022)	Formative	Exercises to address drawing ability, observation ability, and communicating a medical concept through drawing.

Diana (2021)	Formative	Exam scores on shelf exams (I.e., exams about practical learning on a clinical placement).

Loh (2012)	Formative	Submission of five best photographs with case summaries and three poorest photographs, with reflections on weaknesses and what students had learned from the course.


## Discussion

We aimed to identify the countries in which active arts engagement is being used in undergraduate medical education, to understand their purpose(s) (i.e., learning objectives) and whether these objectives are achieved, and to determine how programs of active arts engagement are being evaluated and how learners are being assessed.

We were interested in analyzing articles that involved active arts engagement, and so full-text screening necessarily involved dividing articles into whether engagement with arts was active or receptive. This screening process thus enabled us to compare numbers for active versus receptive engagement, and we observed that studies of active arts engagement were disproportionately low (n = 134) compared to those we excluded because they described receptive engagement (n = 305) (Figure 1). This pattern may signal a missed opportunity to leverage the benefits of arts creation, given the link between active engagement and wellbeing, resilience, self-awareness, and personal growth among medical learners [7,15,16].

### In which countries is active arts engagement being discussed in relation to undergraduate medical education?

We identified 134 studies across 27 countries (Figure 2). However, 88/134 studies were conducted in the USA and UK, 112 studies were conducted in high-income countries and only 17 papers involved international collaboration. Such findings suggest that lower income countries do not have a strong voice in the knowledge conversations, and there is limited collaboration between educators across geographical borders. While this pattern is not unique to arts creation and is illustrated across a range of medical education topics [46], closer collaboration has the potential to conduct scaled research that has increased relevance for international medical educators and learners.

### What purpose(s) (i.e., learning objectives) is active arts engagement serving within undergraduate medical education? Are the learning objectives achieved?

Active arts engagement primarily served the purpose of mastering skills, perspective-taking, and personal insight, with a lesser focus on social advocacy. Most studies reported that the learning objectives were achieved. While encouraging, we must be mindful that researchers may be more likely to share educational interventions resulting in positive outcomes for learners, and so these studies are also more likely to be published [47].

Social advocacy has been defined as the cultivation of civic mindedness and advocating for transformative changes in healthcare and society at large [2]. This requires a deep understanding of social inequities and how these inequities relate to the role and actions—or inactions—of physicians. This conceptualisation of social advocacy closely aligns with the purpose and focus of medical humanities. It is interesting, then, that although a commonly stated focus of the medical humanities is social justice [48], our review reports a lack of engagement with social advocacy as a learning objective. This suggests a potential disconnect between the use of active arts engagement and its operationalization within medical curricula. We suggest that educators and curriculum developers direct attention to how the objectives of active arts engagement programs align, or do not align, with social advocacy, and the conditions required to deliver active arts engagement.

### How are programs of active arts engagement being evaluated?

Based on Kirkpatrick’s model [20], most studies evaluated whether students found the program useful, relevant, or engaging. In some instances, the studies evaluated whether students acquired the intended knowledge or skills. Almost no studies evaluated changes in students’ behavior and whether the outcomes achieved could be attributed only to the program. In addition, the quality of the evaluation in many cases was lacking (e.g., not utilizing validated survey instruments, not collecting pre-and-post intervention data, and not following up or recording dropout rates). A recent narrative review has aimed to bridge this gap by synthesizing quantitative metrics and psychometric scales that can be used for assessing arts programs in medical education [49].

We would like to highlight a tension, in that, whilst the evidence from studies within this review indicates that students learn important skills when engaging actively with the arts, the quality of the evidence base is relatively low. We lack robust approaches to evaluating such programming to then draw meaningful conclusions about student learning. If we are to truly advocate for more active arts engagement in medical education, we should seek to understand students’ learning and changes in their behaviour as a direct result of engaging with the arts. Doing so requires more strategic planning around program design and evaluation, including collaborating in ways that ensure more arts and humanities expertise in this process, as well as more strategic approaches to study design and reporting to build a body of literature with fewer methodological limitations.

### How is active arts engagement integrated into curricula?

Most studies did not use any methods to assess learners. If we accept that, traditionally, assessment is a ‘driver for learning’ and that students focus their efforts on content that is assessed more so than content that is not [50,51], what impact might the lack of assessment be having on the value that students attach to learning through active arts engagement? While we accept that the learning outcomes active arts engagement seeks to achieve are challenging to assess, medical educators have found ways to assess reflective writing, therefore this might also be possible for creative activities [52].

Most interventions were optional, raising questions regarding how valued the arts are in medical education. This devaluing may be perpetuated further when we consider who facilitates these programs: medical faculty. Students often go to labour wards to learn about childbirth from midwives and hospices to learn about end-of-life conversations from palliative care clinicians. This centering of ‘expert’ is not always applied to arts programs in medical education. Only nine medical faculty members across all studies had an arts background. This finding is consistent with Moniz et al. [1] who argued that the predominance of medical faculty teaching and, thus, publishing on this topic has led to an absence of artist-practitioner voices in the literature. We agree and highlight an additional concern: when arts experts taught alongside medical faculty, we noted a wider range of learning objectives. It is therefore worth considering what pedagogical potential is lost when we fail to approach art interventions with the same rigor and standard for expertise as other aspects of medical education.

Still, we recognize the challenges involved in integrating arts interventions into medical training. When we consider the limited budgets of medical schools, especially on an international scale, perhaps it is ‘good enough’ for these interventions to be delivered by medical faculty regardless of their expertise in a particular artistic medium, especially if they possess expertise in facilitating safe conversations that can lead to transformational learning. This may have implications for applicability and access across the world. Most medical students’ skills will likely be at a novice level, and the purpose of the active arts engagement is not to teach art; but rather, to use art as a medium through which to meet learning objectives. The idea that any interested medical faculty can potentially run an active arts engagement program may make its implementation less daunting, thus giving opportunity for art to be interspersed into medical education more widely.

### Limitations

Given that one of our central aims was to explore international perspectives, the primary limitation is that, as a result of resource constraints, we only included articles published in English, whereas relevant articles have certainly been published in other languages. In addition, we did not search the grey literature, which might have also helped identify articles from a wider range of countries, who may not have easy access or financial resources to publish in journals. Future work should seek funding for translation where necessary.

Furthermore, there is a likelihood of human error and selection bias considering that the previous scoping review had slightly different eligibility criteria. As we used the same database-specific subject headings as in the previous scoping review, it is also possible that some relevant studies might have been missed due to the use of older subject headings.

Most studies reviewed lacked validity and reliability, in the absence of adequate evaluation and/or reliable outcome measures, so results should be interpreted accordingly. Furthermore, due to publication bias, low intensity and/or less successful initiatives are unlikely to get published. As such, we may be missing important information regarding the practical challenges of integrating such programs into medical curricula.

## Conclusion

This scoping review synthesised active arts engagement programming in medical education internationally to understand the evidence-base and how such programs are incorporated into medical curricula.

Our study included 134 studies across 27 countries, most of which were high-income. In our dataset, most programming served the purpose of mastering skills, perspective-taking, and personal insight. Studies focusing on social advocacy were lacking, which is concerning considering that social justice is a key focus of medical humanities to realize the physicians’ role in addressing health inequalities. Program evaluation and learner assessment were also lacking in the studies analyzed. Furthermore, studies of active arts engagement were disproportionately low compared to receptive engagement, signaling missed opportunities to leverage the benefits of arts creation.

To avoid devaluing the arts in medical curricula, we suggest that medical educators: a) direct attention to creative opportunities to engage students with social advocacy; b) collaborate with arts/humanities professionals and international medical educators; and c) consider more meaningful and strategic integrations of active arts engagement into medical curricula, approaching them with the same rigor as other medical education programs to maximize their pedagogical potential.
